# Supplementary material for: Joint assessment of density correlations and fluctuations for analysing spatial tree patterns
Source: R Soc Open Sci. 2021 Jan 20;8(1):202200. doi: 10.1098/rsos.202200 (PMC7890483; doi:10.1098/rsos.202200)
Supplement: Supplementary Information [file rsos202200supp1.pdf]

# Supplementary Information: Joint assessment of density correlations and fluctuations for analysing spatial tree patterns

**Villegas, P.**[1,*]**, Cavagna, A.**[1,2]**, Cencini, M.**[1]**, Fort, H.**[3]**, and Grigera, T.S.**[1,4,5,6]

[1]Istituto dei Sistemi Complessi, Consiglio Nazionale delle Ricerche, via dei Taurini 19, 00185 Rome, Italy
[2]Dipartimento di Fisica, Università Sapienza, 00185 Rome, Italy
[3]Institute of Physics, Faculty of Science, Universidad de la República, Iguá 4225, Montevideo 11400 Uruguay
[4]Instituto de Física de Líquidos y Sistemas Biológicos — CONICET and Universidad Nacional de La Plata, La Plata, Argentina
[5]CCT CONICET La Plata, Consejo Nacional de Investigaciones Científicas y Técnicas, Argentina
[6]Departamento de Física, Facultad de Ciencias Exactas, Universidad Nacional de La Plata, Argentina
[*]Correspondence and requests for materials should be addressed to pvillegas@ugr.es

## SI-1: Identification of the border with alpha shapes

As discussed in the main text, the statistical estimators able to avoid the biases induced by the borders can only be applied once these have been identified. Finding the borders of a set of points is thus a crucial issue. In the main text we discuss a powerful method to define the (internal and external) perimeter of a set of points.

A first approximation to the borders is to consider the Convex Hull (CH) of the set of points. With simple algorithms one can find the list of points conforming the convex envelope of the system. For most species in the BCI census, this essentially corresponds with the edges of the rectangular area of the plot. While finding the CH of a set of points is typically fast, field data are in general not convex. A clear example of such a problem consists of an hypothetical spatial distribution of trees surrounding an empty area, e.g. the forest glade exhibited by *H. prunifolius* (Fig. 2 of main text). In a case like this, besides the biases on neighbour counting induced by the presence of the void space, the CH method overestimates the covered area, and consequently underestimates the mean density.

As explained in the main text, a better determination of the borders can be achieved using the so-called $\alpha-$shape method (AS). This procedure is able to delineate both the internal and external edges – which are neither necessarily convex nor connected– of the system by filling it with discs of radius $\alpha$ (a tuning parameter of the algorithm, which in general is not possible to fix in an objective way).

Border points are identified by considering all point pairs that can be touched by an empty disc of radius alpha. Schematically, the algorithm works as follows: (i) Once two points have been selected (black circles in Fig. SI.1), two circles of radius $\alpha$ are centered at each point, calculating the intersection points of the two circles (red points in Fig. SI.1). (ii) From the two intersection points of the circles, draw two new circles of radius $\alpha$ (red circles in Fig. SI.1), which by construction contain the black points. (iii) If there are no points inside one of the circles, black points are considered border points. Specifically,

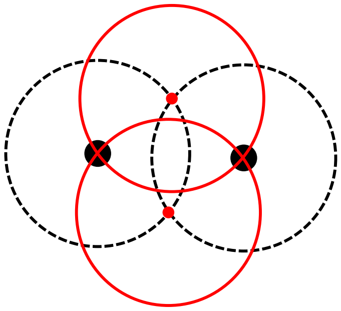

**Figure SI.1.** Two discs 'hitting' the black points can be plotted. If either of the new circles contains no points in one of such red discs, black points are considered like border points.

to compute the borders with $\alpha$-shape we used a dedicated and efficient Matlab library,[†] and computed the covered area via Delaunay triangulation, which is also implemented in the same library.

The remaining problem is now the choice of the free parameter $\alpha$. If the value of $\alpha$ is too large we get back the convex hull, while if it is too small, all the discs will penetrate into the distribution of points, breaking it up completely and producing a collection of small sets of points. Given these limiting cases, typically a good way of finding an appropriate value of $\alpha$ is to study the behaviour of the covered area (or, equivalently, of the density) as a function of $\alpha$. Thus, when the concavities of different sizes are eliminated, a sudden jump of the area is expected, followed by a plateau which finally yields a good estimation $\alpha$ small enough but larger than the typical first-neighbour distance. As an example, in the inset of Fig. SI.2a we show the behaviour of the total covered area, estimated via Delaunay triangulation[1], for a set of Poisson-distributed points in a square domain of side $L = 20$ where we removed two semicircles of radius $r_1 = 3$ and $r_2 = 8$. As clear from the plot, below a value of $\alpha \approx r_1$ the covered area converges to the analytical value, $L^2 - \pi^2(r_1^2 + r_2^2)/4$, provided the number of points is large enough.

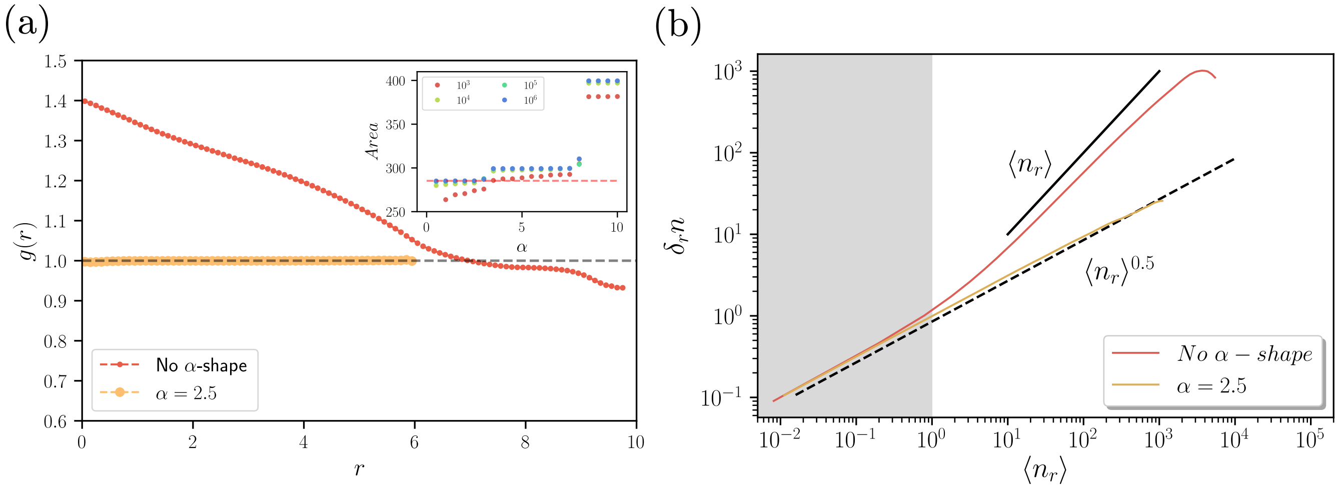


**Figure SI.2.** Effects of borders on density correlation and fluctuations. For a set of $N = 10^5$ points Poisson-distributed in a $20 \times 20$ square with two empty semicircles of radii $r_1 = 3$ and $r_2 = 8$ we compute (a) the pair correlation function $g(r)$ and (b) Taylor's law, using the borders as defined by the CH (red) or $\alpha-$shapes for $\alpha = 2.5$ (orange). Black dashed line stands for theoretical results, $g(r) = 1$ and $\delta_r n_r = \langle n_r \rangle^{1/2}$. Inset of (a): covered area versus $\alpha$ for different number of points included in the same set as main panel. Notice that the covered area $A_\alpha$ converges to the analytical value (dashed line) for $\alpha < 3$ when the number of points $N$ is large enough (in this case $N \gtrsim 10^4$).

The main panel of Fig. SI.2a shows the effects of using the different boundaries on the computation of the pair correlation function (PCF), $g(r)$. When using the convex hull, $g(r)$ is found to be greater than 1 at small scales ($r < 6$) and converge to $\approx 1$ at large scales. This is a spurious "clumping" effect due to an incorrect choice of borders. In contrast, when using $\alpha$-shape, the expected result $g(r) = 1$ is found.

In Fig. SI.2b we show how biases induced by the incorrect identification of the borders impact on the evaluation of the density fluctuations, i.e. Taylor's law (TL). Without $\alpha$-shapes, i.e. using the borders of the square, one would have found strong deviations of TL exponent from the Poisson value $\gamma = 1/2$. Indeed, when failing to exclude the empty area we observe a power law with exponent $\gamma \approx 1$, which via Eq. (3.3) of main text would imply fluctuations that do not decay with the scale of observation. Instead, with the proper borders (i.e. removing the empty semicircles from the computation), and using the Hanisch method for avoiding border bias, we obtain the expected exponent $\gamma = 1/2$. The main reason for such spurious results is that empty cells are those which typically increase the fluctuations, so one should ensure, at each scale $r$, to include only the empty or semi-empty cells which really belong to the set of points. We conclude observing that at very small scales (gray shaded area in Fig. SI.2b) $\gamma = 1/2$ also with the wrong borders provided $\langle n_r \rangle < 1$, this is the trival regime discussed in Sec. 3 of the main text.

### SI-1.1: Area and density estimation in BCI using the $\alpha$-shapes

The dependence of covered area or density with $\alpha$ is not always as clear as in the inset of Fig. SI.2a. In actual BCI data many scales can be involved, and the step-like behaviour of the covered area is more an exception than a rule. This is particularly

[†] https://it.mathworks.com/help/matlab/ref/alphashape.html

clear from Figs. SI.3a-b where we show, for *H. prunifolius* and *T. panamensis* the dependence on $\alpha$ of the fraction of empty area $(A - A_\alpha)/A$ (where $A$ and is the area of the rectangular BCI plot and $A_\alpha$ that determined with the $\alpha$-shape algorithm). We observe an almost continuous and monotonic decay with $\alpha$ until the CH limit, in which no concavities are identified. Figures SI.3c-d show that the same applies to the relative difference between the $\alpha$-shape estimated density $\rho_\alpha = N/A_\alpha$ and the naive estimation, $\rho_0 = N/A$.

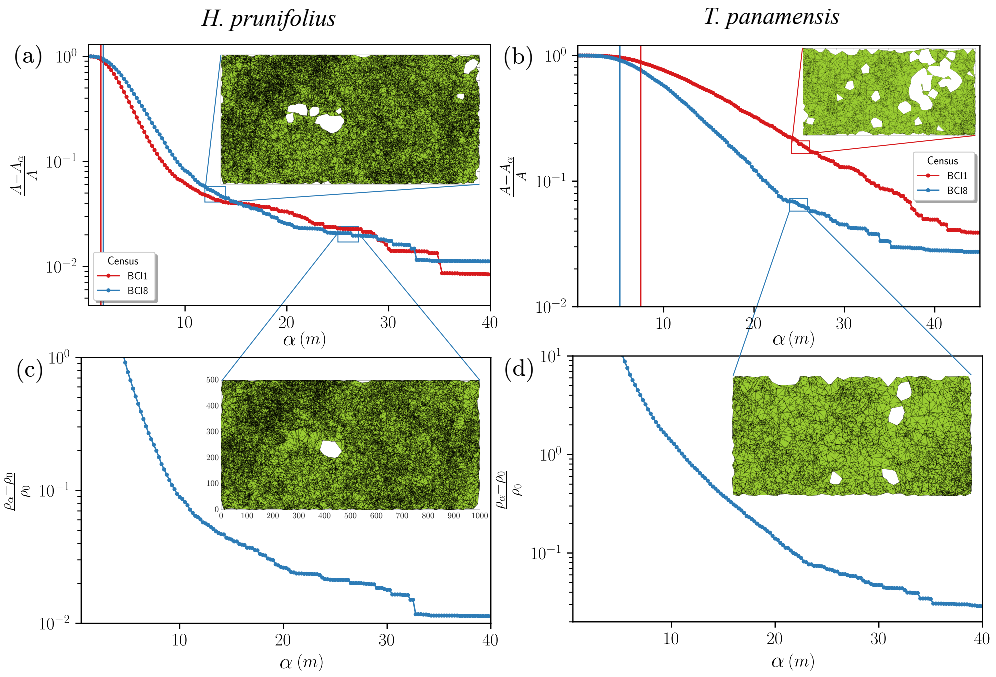


**Figure SI.3.** Estimation of covered area and density with $\alpha$-shapes. (a,b) Fraction of empty area $(A - A_\alpha)/A$ vs. $\alpha$, where $A$ is the area of the entire BCI plot and $A_\alpha$ the area with the concavities eliminated via $\alpha$-shape. (c,d) Relative difference between the naive density estimation $\rho_0$ and the density $\rho_\alpha$ computed using the area obtained with $\alpha$-shape vs. $\alpha$. Panels (a,c) correspond to *H. prunifolius* and (b,d) to *T. panamensis*. Note that no clear plateau can be identified (compare with the inset of Fig. SI.2a). For the fraction empty area we show results for the first census (red points) and the last one (blue points). Vertical bars indicate the interparticle distance for each census. Insets (a, c): area covered by *H. prunifolius* in census 8 for two values of $\alpha$ larger than the interparticle distance. Insets (b,d): area covered by *T. panamensis* for the same value of $\alpha$ in census 1 (b) and census 8 (d). The covered area depicted in the inset and its numerical value were obtained with Delaunay triangulation.

In the absence of clear plateaus, one is forced to use a more subjective criterion to fix the value of $\alpha$. Consider for instance the difference in covered area for two different values of $\alpha$ for *H. prunifolius* in the insets of Figs. SI.3a,c. To choose $\alpha$ we proceeded as follows: first, we always required $\alpha$ to be significantly larger than the mean distance between nearest-neighbour trees, to avoid fragmentation of the system (otherwise the covered area would be unreasonably small, see Figs. SI.3a-b). Inter-particle distances are plotted with vertical bars in the main panels of Figs.SI.3a-b. Second, we have taken $\alpha$ small enough to assure the identification of empty areas in the spatial distribution of different species, which were always checked by visual inspection. Further, to be more systematic, we also selected $\alpha$-values when the continuous decay of the estimated area changes its curvature.

As seen in the insets of Fig. SI.3, for *H. prunifolius* (see also Fig. 2 of main text) and *T. panamensis* such criterion provides a reasonable estimation of the covered area. In particular, for *H. prunifolius* our choice identified the empty region depicted in Fig. 2 of the main text (and in the inset of Fig. SI.3a) which actually corresponds to swampland region unfavorable to the establishement of this species[2]. Unfortunately, for other species we do not have additional information of this kind to check the validity of the choice of $\alpha$. Comparing the insets of Figs. SI.3b,d corresponding to the area covered by *T. panamensis* for the same value of $\alpha$ but for the first and last census, one can appreciate how some of the holes present in census 1 were filled in census 8, meaning that this species experienced an expansion in between the two censuses, whose consequences for the PCF are discussed in the main text (in particular Fig. 4d).

In Fig. SI.4 we show the border trees of the tree patterns for the ten most abundant species in BCI (which account for about half of all the trees present) together with the value of $\alpha$ we used. Finally, Fig. SI.5 shows the evolution of the borders in three different censuses for the contracting species *P. cordulatum* and *P. armata*. Notice that *P. cordulatum* seems to loose

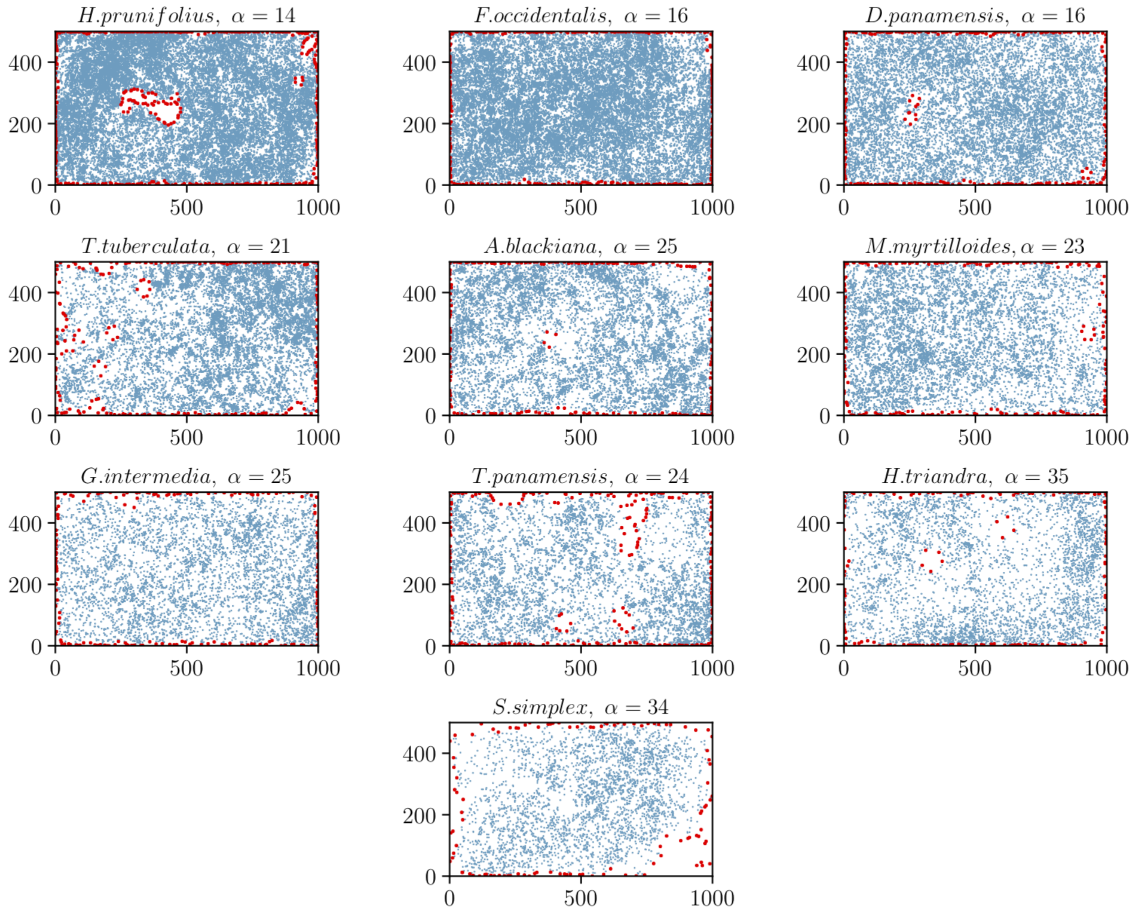


**Figure SI.4.** Border trees (red) identified with $\alpha$-shapes for the given values of $\alpha$ for the ten most abundant species in BCI.

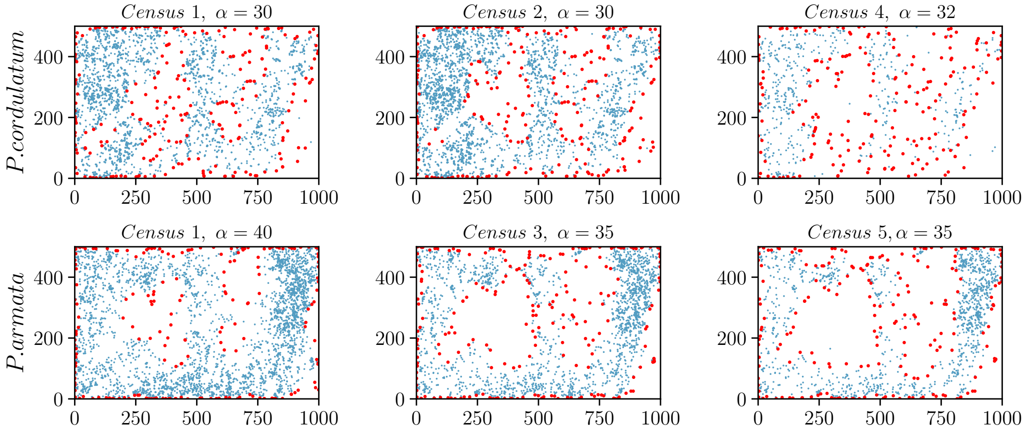


**Figure SI.5.** Border trees (red) identified with $\alpha$-shapes for the given values of $\alpha$ for two contracting species *P. cordulatum* (upper panels) and *P. armata* (lower panels) in different censuses.

structure (and as time progress looks more and more homogeneous), unlike *P. armata.* This is reflected in the behavior of density correlations (Fig. 5 of main text) and fluctuations (Fig.SI.8 and SI.9).

## SI-2: Density correlations and fluctuations at the single species level

As discussed in the main text, the most abundant species in Barro Colorado, while coherently showing evidence of clumping at small scales ($g(r) > 1$), clearly display very different behaviours at large scales. In the main text we mainly discussed three representative species. Here, we analyze those of Fig. SI.4, examining the effects of using $\alpha$-shapes on both the PCF (Fig. SI.6) and Taylor's law (Fig. SI.7).

Figure SI.6a shows the PCF computed using the rectangular plot borders. For all species we observe clumping at short distances, but at larger scales behaviours range from clear anticorrelation ($g(r) < 1$) at intermediate distances (see e.g. *H. triandra*), to species displaying a large scale plateaux at values either larger than 1 (e.g. *F. occidentalis* and *S. simplex*) or very near 1 (as e.g. *H. prunifolius* and *G. intermedia*). When using $\alpha$-shapes to find the border, as shown in Fig. SI.6b, some of the species recover a plateaux to 1 *(T. panamensis)* and anticorrelations essentially disappear, but still a quite large variability in the large scale behavior persists for other species.

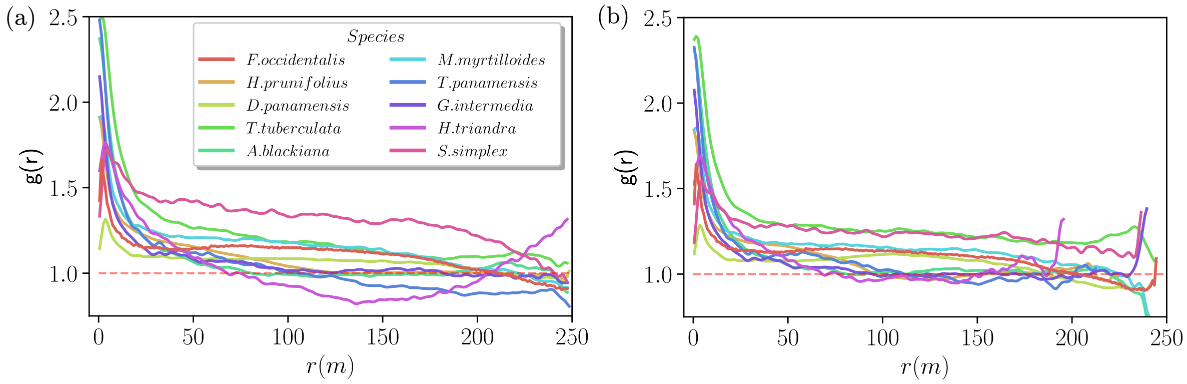


**Figure SI.6.** Pair correlation function $g(r)$ computed without (a) and with (b) $\alpha$-shapes for the ten most abundant species in BCI (distinguished by colour). The values of $\alpha$ used for each species and the resulting borders can be read in Fig. SI.4. Red dashed line shows the theoretical expectation $g(r) = 1$ for a completely random distribution of points.

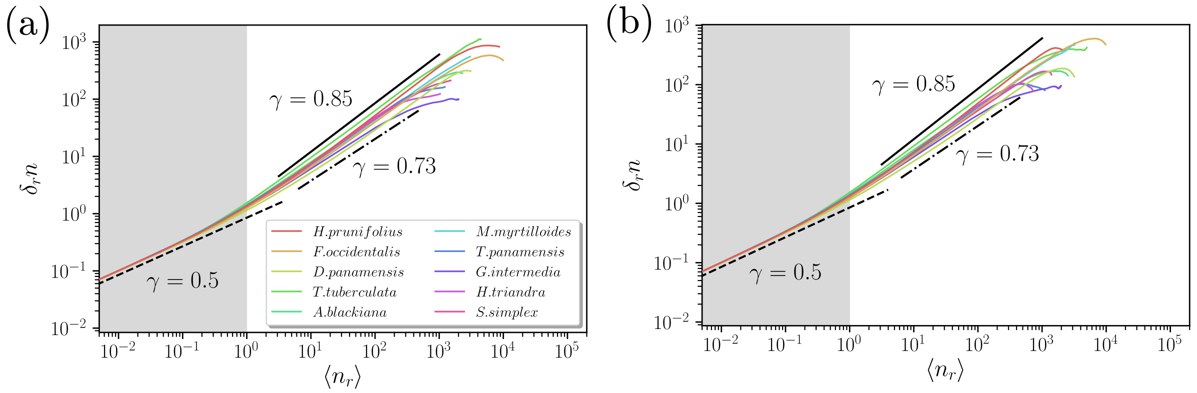


**Figure SI.7.** Density fluctuations measured via Taylor's law, i.e. looking at how the root mean square deviations of number of trees in cells of size $r$, $\delta_r n$, changes with mean $\langle n_r \rangle$, without (a) and with (b) $\alpha$-shapes. Dashed lines are the theoretical expectation, $\gamma = 1/2$, for a completely random process, while the solid and dashed-dotted ones bracket the (fitted) limiting value of the exponent $\gamma$. The grey shaded area indicates the region of scales below the mean distance between neighbouring trees, where $\langle n_r \rangle < 1$.

The fact that anti-correlations disappear means that some voids have been removed (this is likely the case of *H. triandra,* see e.g. Fig. SI.4). As briefly discussed in the main text, this points out a delicate issue when using methods such as $\alpha$-shapes to find borders: anticorrelations are not always spurious features of a tree distribution, so that when removing them one should be aware that one might be masking a genuine process responsible for them. On the other hand, in cases such as *T. panamensis,* which displays mild anticorrelation at large distances in Fig. SI.6a (see also main text Fig. 4d), the most likely explanation is that the species is experiencing an expansion (see also the insets of Fig. SI.3b-d).

Moving on to density fluctuations via Taylor's law (Sec. 3 of main text), we show in Fig. SI.7 how the root mean square deviations of number of trees in cells of size $r$, $\delta_r n$, changes with mean $\langle n_r \rangle$. We observed that apart from small inessential quantitative changes, the TL is not sensitive to using $\alpha$-shapes or not. This is somehow surprising for *H. prunifolius,f* for which a large hole in the domain is present. As for the quantitative aspects, we found that besides the trivial convergence to $\gamma = 1/2$ below the interparticle distance (gray shaded area), at large scales the behavior is always anomalous ($\gamma > 1/2$) for all species, with $\gamma$ varying in the range $[0.73 : 0.85]$. The effect of using $\alpha$-shapes or not is to produce small changes in the exponent of the single species, but the range of values remains basically the same.

In Figs. SI.8 and SI.9, we show density fluctuations for the two contracting species *P. cordulatum* and *P. armata*, showing the effect of using $\alpha$-shapes. We observe different behavior for the two species. For *P. cordulatum* we find strong dependence on the definition of the border, especially in census 4. In particular, $\gamma$ seems to approach the value $1/2$ indicative of a tendency of recovery of homogeneity. This seems to be confirmed by the behavior of the PCF shown in Fig.5a of the main text and with the visual impression from the top panels of Fig. SI.5, which show a qualitative change of the tree distribution in census 4 with respect to the previous two. For *P. armata* we found that $\gamma$ changes from 0.9 to 0.85, but there is no tendency to approach a homogeneous process.

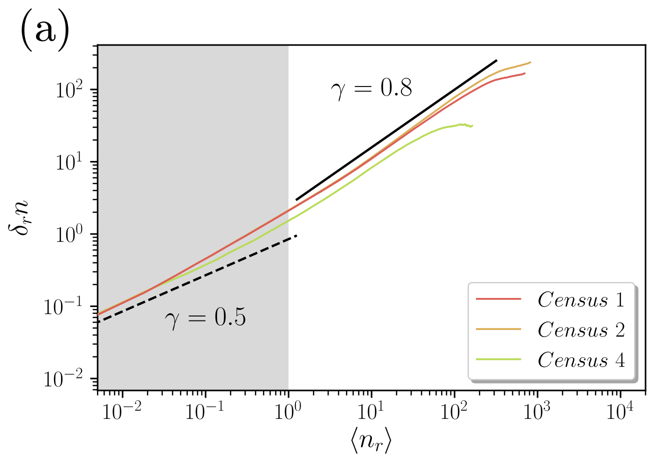


**Figure SI.8.** Taylor's law computed without (a) and with (b) $\alpha$-shapes for *P. cordulatum* in censuses 1 (with $\alpha = 30$), 2 ($\alpha = 30$) and 4 ($\alpha = 32$). Lines indicate the slopes as labeled. The grey shaded area indicates the region of scales below the mean distance between neighbouring trees, where $\langle n_r \rangle < 1$.

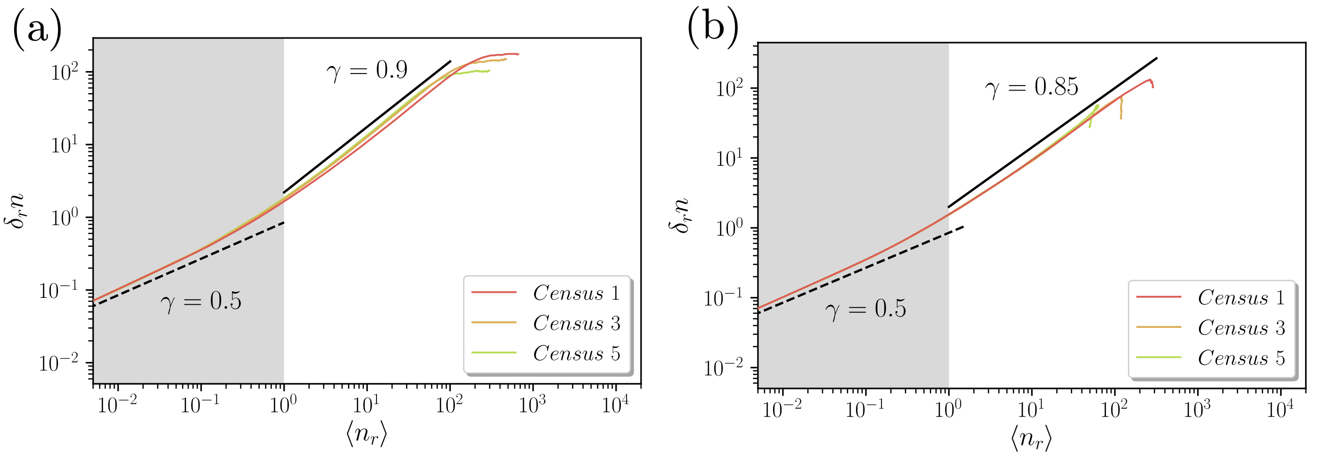


**Figure SI.9.** As Fig. SI.8 but for *P. armata* in censuses 1 ($\alpha = 40$) , 3 ($\alpha = 35$) and 5 ($\alpha = 35$).

## SI-3: Dual representation of the spatially explicit neutral model

The multispecies voter model has been simulated exploiting its duality with a system of coalescing random walkers with a killing rate[3–5]. The main advantage of this method is that it allows for very fast simulations to produce independent realization of sample patterns at the (non-equilibrium) stationary state virtually free from boundary effects such as that can be introduced by periodic boundary conditions (typically employed when simulating in the forward representation described in the main text).

The dual process is built as follows. At the beginning, each lattice site is filled with a random walker. The dual process proceeds backwards in time to reconstruct the ancestry of the species. At each discrete (backward) time step, with probability $1 - \nu$, a randomly chosen walker is moved to a different site (which can be outside the sampled domain, since the lattice is infinite though we only observe a finite portion), chosen according to a distribution which depends on the distance from the original site $r$ —i.e. the dispersal kernel $P(\boldsymbol{r})$, which we have chosen to be Gaussian centred in the original site and with standard deviation $\sigma$. If the landing site is occupied, the two walkers coalesce and one of them is removed, keeping trace of the coalescing partner. With complementary probability $\nu$, the randomly chosen walker is killed. This corresponds to a speciation event in the forward in time description. The simulation proceeds until only one walker is present. Finally, having stored the whole tree of coalescences and knowing which walker was killed one can trace back the entire genealogical tree of a species up to the speciation event that originated it. Then, having labelled each walker in such a way to be able to identify its initial position, one can assign to each site of the lattice. Since the number of walkers decreases at each coalescence or killing event, the simulation time becomes faster as time proceeds. Of course, this procedure can be used only if one is interested in the static, long-term, properties of the model.

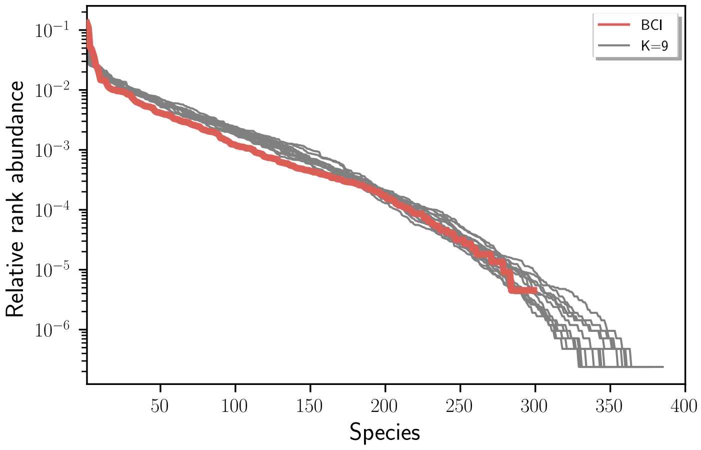


**Figure SI.10.** Rank abundance curves (grey lines) for different realizations of the MVM with $\sigma = 9$, $\nu = 3.8 \cdot 10^{-6}$ and the rank abundance of the 8th census of Barro Colorado (red line).

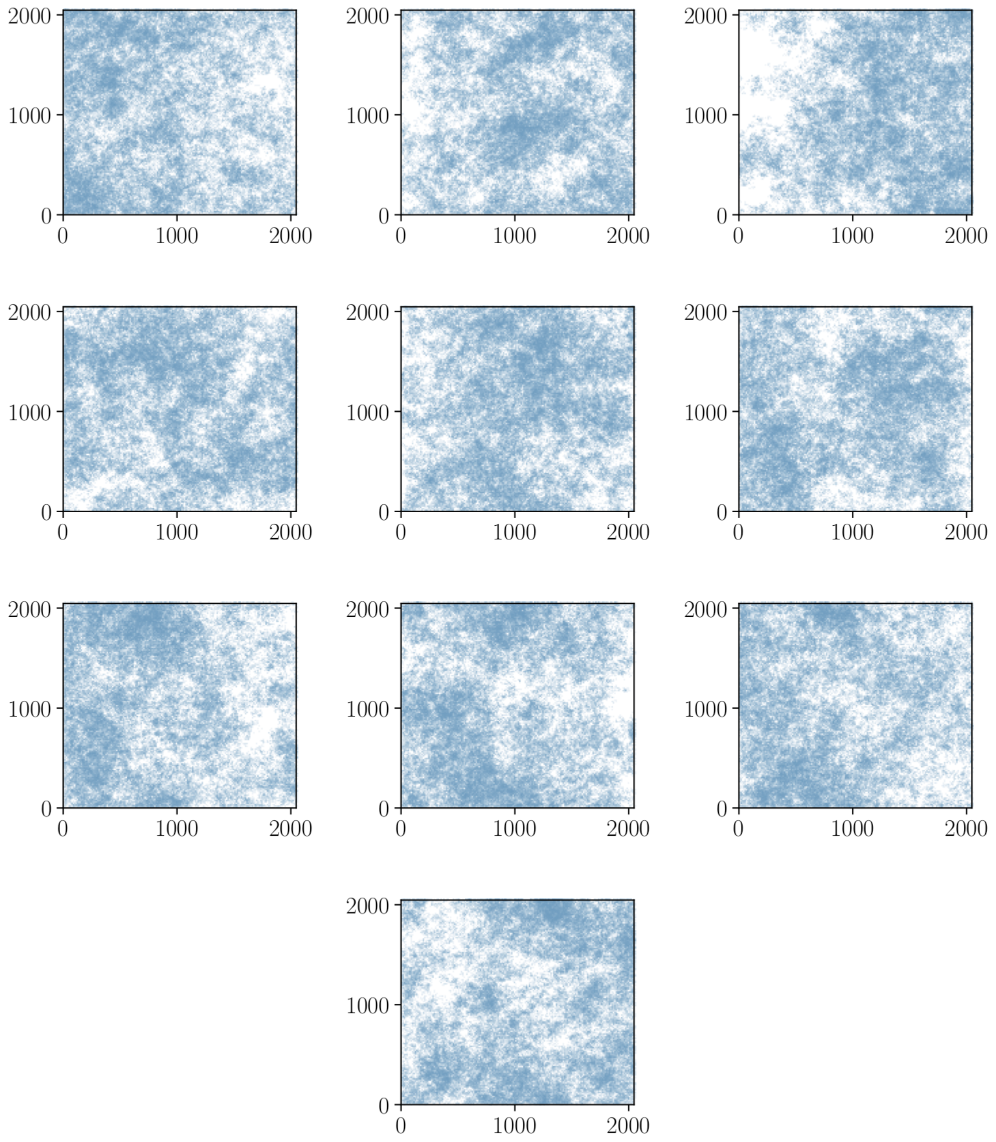


**Figure SI.11.** Spatial patterns for different species in the MVM. Parameters: $\sigma = 9$, $\nu = 3.8 \cdot 10^{-6}$ and $N_i \in (142000 - 157000)$.

### SI-3.1: Effects of $\alpha - shapes$ method

In the case of the above described spatially explicit neutral model we have a complete epistemic knowledge of the system and, in particular, we know that the (eco-)system is homogeneous in the sense that all points occupied by trees are equivalent a priori. This means that there are no reasons to consider borders besides the square where the process is simulated, which should be regarded as a sample of an infinite system. However, as clear from Fig. SI.11 voids are present due to the competition between species. Thus, for the sake of completeness and comparison with BCI, it is worth considering the effect of $\alpha$-shapes also within the patterns generated with the neutral model.

In Fig. SI.12 we show the border of the spatial patterns for some selected species in a neutral environment together with the value of $\alpha$ we used.

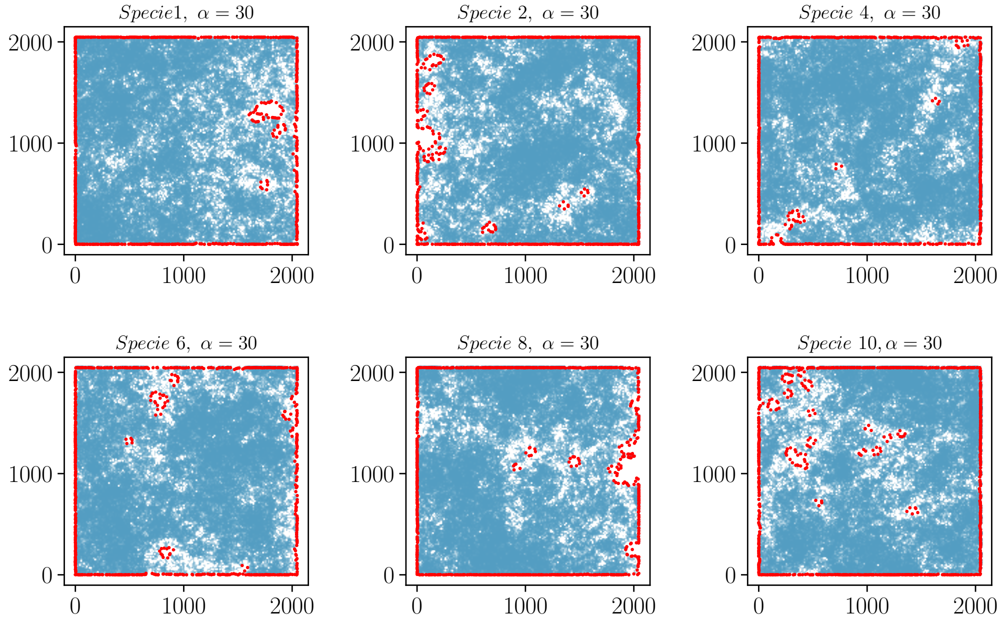


**Figure SI.12.** Spatial distribution for some selected simulated species (with $N_i \approx 1.4$–$1.6 \cdot 10^5$ trees) with the neutral model in a lattice of side $L = 2048$. Borders are outlined in red. The internal borders can be clearly appreciated when $\alpha - shapes$ are employed.

Regarding to the g(r), some differences between both cases (see Fig.SI.13a and Fig.SI.13b) can be observed. Firstly, the behavior at short scales is not affected by the $\alpha$-shape method. That is, clumping behavior is completely robust against changes of the borders. For larger scales, the effect of $\alpha - shapes$ consists of mainly removing anticorrelations. However, we stress again, in this case there is no plausible reason –as also discussed above and in the main text– to remove such anticorrelations in the pattern generated by this model.

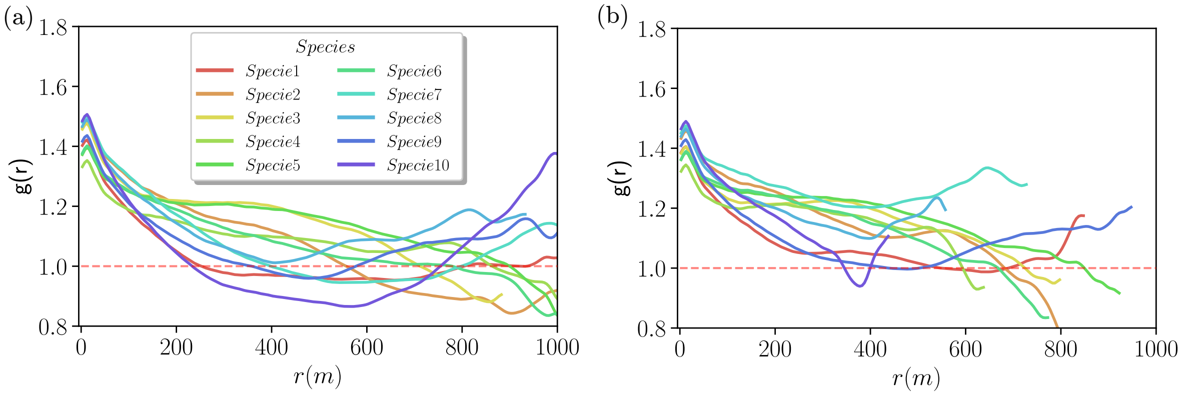


**Figure SI.13.** Pair correlation function for ten selected simulated species with the neutral model (a) without $\alpha - shapes$ and (b) employing the $\alpha - shape$ method with a predefined radius $\alpha = 30$. For some of the species anticorrelations at mid-scales clearly disappears (e.g. Specie10).

For the sake of completeness, we also investigated the possible role of boundaries in the behavior of spatial density fluctuations, i.e. in the behavior of the Taylor's law. As shown in Fig.SI.14a and Fig.SI.14b, we have not found any bias on the value of the exponent $\gamma$ when the $\alpha - shape$ method is employed. Thus, we can safely say that –for reasonable values of $alpha$, larger than the interparticle distance– large spatial fluctuations do not depend on the selection of the borders for the neutral model, at least for the most abundant species there represented.

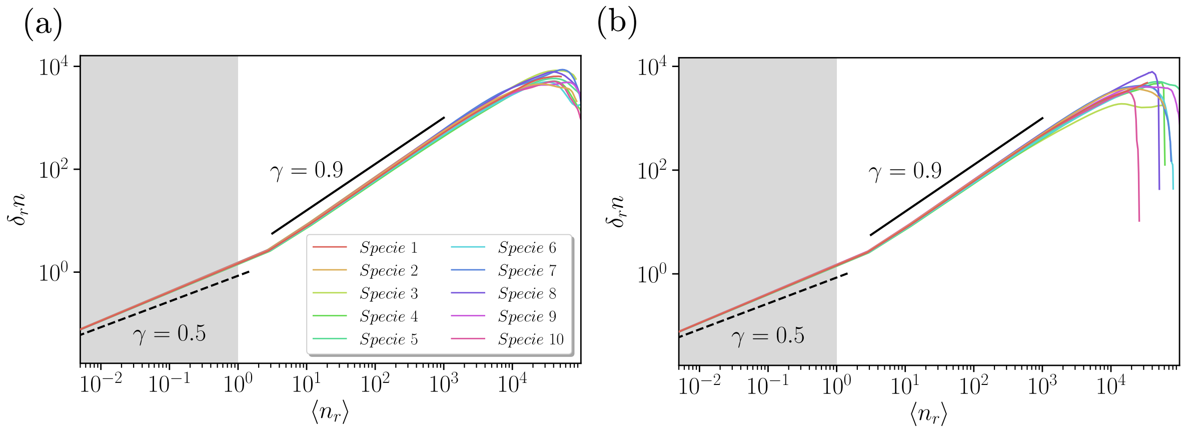


**Figure SI.14.** Spatial density fluctuations for the ten selected simulated species with the neutral model (a) without $\alpha - shapes$ and (b) employing the $\alpha - shape$ method with a predefined radius $\alpha = 30$. There is no qualitatively change between both cases.

## References


1. Delaunay, B. *et al.* Sur la sphere vide. *Izv. Akad. Nauk SSSR, Otdelenie Matematicheskii i Estestvennyka Nauk* **7**, 1–2 (1934).
2. Horvát, S., Derzsi, A., Néda, Z. & Balog, A. A spatially explicit model for tropical tree diversity patterns. *J. Theor. Biol.* **265**, 517–523 (2010).
3. Bramson, M., Cox, J. T. & Durrett, R. Spatial models for species area curves. *Annals Probab.* **24**, 1727–1751 (1996).
4. Durrett, R. & Levin, S. Spatial models for species-area curves. *J. Theor. Biol.* **179**, 119–127 (1996).
5. Holley, R. A. & Liggett, T. M. Ergodic theorems for weakly interacting infinite systems and the voter model. *Annals Probab.* 643–663 (1975).
